# Perioperative myocardial injury in revascularized coronary patients who undergo noncardiac surgery

**DOI:** 10.1371/journal.pone.0219043

**Published:** 2019-06-27

**Authors:** Seung-Hwa Lee, Myung Soo Park, Young Bin Song, Jungchan Park, Jaeyoun Kim, Sangmin Maria Lee, Young Tak Lee

**Affiliations:** 1 Department of Medicine, Heart, Stroke and Vascular Institute, Samsung Medical Center, Sungkyunkwan University School of Medicine, Seoul, Republic of Korea; 2 Department of Medicine, Dongtan Sacred Heart Hospital, Hwasung, Hallym University School of Medicine, Republic of Korea; 3 Department of Anesthesiology and Pain Medicine, Samsung Medical Center, Sungkyunkwan University School of Medicine, Seoul, Republic of Korea; 4 Department of Thoracic and Cardiovascular Surgery, Samsung Medical Center, Sungkyunkwan University School of Medicine, Seoul, Republic of Korea; Azienda Ospedaliero Universitaria Careggi, ITALY

## Abstract

**Background:**

Whether high-sensitivity cardiac troponin elevation during the perioperative period is associated with poor clinical outcome in revascularized coronary patients who undergo noncardiac surgery remains unclear. We investigated the effects of perioperative troponin elevation on the long-term clinical outcomes of patients with a history of coronary revascularization.

**Methods:**

We analyzed patients whose pre- or postoperative high-sensitivity cardiac troponin I (hs-cTnI) assay results were available. Patients were divided into two groups according to hs-cTnI levels. The patient groups were analyzed separately according to whether hs-cTnI was assessed preoperatively or postoperatively. The primary outcome was all-cause death during the follow-up period.

**Results:**

Median follow-up duration was 25 months (interquartile range 11–50). In the propensity-matched analysis, the risk of all-cause death during follow-up was higher in the group with elevated hs-cTnI group than in the normal group (12.7% vs 6.3%; hazard ratio [HR], 2.67; 95% confidential interval [CI], 1.04–6.82; *p* = 0.04). In the propensity-matched analysis of preoperative hs-cTnI levels, we found no significant difference between the groups in the rate of all-cause death (12.9% vs. 11.9%; HR, 1.06; 95% CI, 0.45–2.50; *p* = 0.89). In the postoperative propensity-matched analysis, all-cause death was higher in patients with elevated hs-cTnI than in those with normal levels (14.9% vs. 5.9%; HR, 2.80; 95% CI, 1.01–7.77; *p* = 0.048).

**Conclusion:**

In revascularized coronary patients who underwent noncardiac surgery, postoperative (but not preoperative) hs-cTnI elevation was associated with all-cause death during follow-up. Larger datasets are needed to support this finding.

## Introduction

Since the development of the high-sensitivity cardiac troponin assay, cardiac troponin analysis has been extended to predict prognosis as well as to diagnose myocardial damage [1,2]. Consequently, troponin testing is used as a screening test in a broad spectrum of non-coronary artery diseases, including end-stage renal disease, sepsis, and congestive heart failure, all of which are associated with elevated troponin levels [3,4]. Broad spectrum usage has also enabled the cardiac troponin assay to provide prognostic information about all-cause mortality during the perioperative period among patients undergoing noncardiac surgery [5,6].

Several studies have shown that perioperative myocardial injury (PMI) before and after noncardiac surgery is associated with short- and long-term mortality [7–13]. However, this hypothesis has not been evaluated in patients with a history of coronary revascularization in the form of percutaneous coronary intervention (PCI) or coronary artery bypass graft surgery (CABG). Moreover, there is limited data about the association between high troponin concentrations and incidents of myocardial injury in patients with stable coronary artery disease [14]. In this study, we sought to evaluate the association between increased high-sensitivity cardiac troponin I (hs-cTnI) levels during the perioperative period and all-cause mortality in revascularized coronary patients who underwent noncardiac surgery.

## Methods

### Study population and data collection

This study is a single-center retrospective study. We initially enrolled 516 patients who underwent noncardiac surgery between June 2010 and June 2015. The inclusion criteria were: 1) patients who had a history of coronary revascularization by either CABG or PCI, 2) patients who underwent noncardiac surgery, and 3) patients whose hs-cTnI levels were included in a pre- or postoperative laboratory evaluation. The exclusion criteria were: 1) patients within 6 months of coronary revascularization and 2) patients who underwent prophylactic CABG or PCI due to coronary artery disease incidentally diagnosed during the preoperative evaluation. In patients with multiple noncardiac surgeries, only the first surgery after revascularization was included in the analysis. Clinical, laboratory, and outcome data during the follow-up period were collected by a trained study coordinator using a standardized case report form according to the study protocol. All participants were analyzed anonymously, and consents were waived by Institutional Review Board (IRB No. 2017-01-026-002) of Samsung Medical Center (Seoul, Republic of Korea).

### Perioperative hs-cTnI level measurement

The hs-cTnI analysis was performed during pre- or postoperative evaluation in patients with established coronary artery disease. Postoperative hs-cTnI elevation was defined as an elevated level in the first 2 days after surgery. Using a highly sensitive immunoassay, cardiac troponin I was measured with an automated analyzer (Advia Centaur XP, Siemens Healthcare Diagnostics, Erlangen, Germany). The lower limit of detection was 0.006 ng/mL, and the upper limit of normal was 0.04 ng/mL, which is the 99th percentile reference provided by the manufacturer [15].

### Definitions and outcomes

Surgery was defined as any procedure performed under general or regional anesthesia. The surgical risk was stratified according to the 2014 European Society of Cardiology/Anesthesiology guidelines [16]. Clinical characteristics and medication histories were collected at admission for surgery. Diabetes mellitus was defined as having a history of type 1 or type 2 diabetes mellitus with a history of treatment by medication or dietary changes. Hypertension was self-reported by the patient or a systolic blood pressure >140 mm Hg at rest. The primary outcome was defined as all-cause death during follow-up.

### Statistical analysis

For continuous variables, differences between groups were compared using a t-test or the Wilcoxon rank-sum test, and the results are presented as the mean ± standard deviation or median and interquartile range (IQR), as applicable. Chi-square or Fisher’s exact tests were used for categorical data. Survival curves were constructed using Kaplan-Meier estimates and compared using the log-rank test. The adjusted hazard ratio (HR) in the preoperative analysis group was compared using a Cox regression based on the following covariates: being male; having diabetes, chronic kidney disease, or an ejection fraction <50%; and using a statin. The adjusted HR of the postoperative analysis group was calculated with the following covariates: having diabetes, chronic kidney disease, an ejection fraction <50%, or multivessel disease and using a statin. To reduce treatment-selection bias and potential confounding factors, we performed rigorous adjustment for baseline and lesion characteristics of the patients using their propensity scores, which we estimated using multiple logistic-regression analyses. Model discrimination was assessed with c-statistics, and model calibration was assessed with Hosmer-Lemeshow statistics. An absolute standardized difference <10% for each measured covariate suggested an appropriate balance between the groups. We compared the continuous variables with paired Student’s t-tests or the Mann-Whitney test as appropriate, and categorical variables were compared with the McNemar or Bhapkar test of symmetry, as appropriate. In the propensity score-matched population, we compared the HRs for outcomes using a stratified Cox regression model. Statistical analyses were performed with R 3.4.3 (R Foundation, Vienna, Austria). All tests were 2-tailed, and *p* < 0.05 was considered statistically significant.

## Results

**Fig 1:**
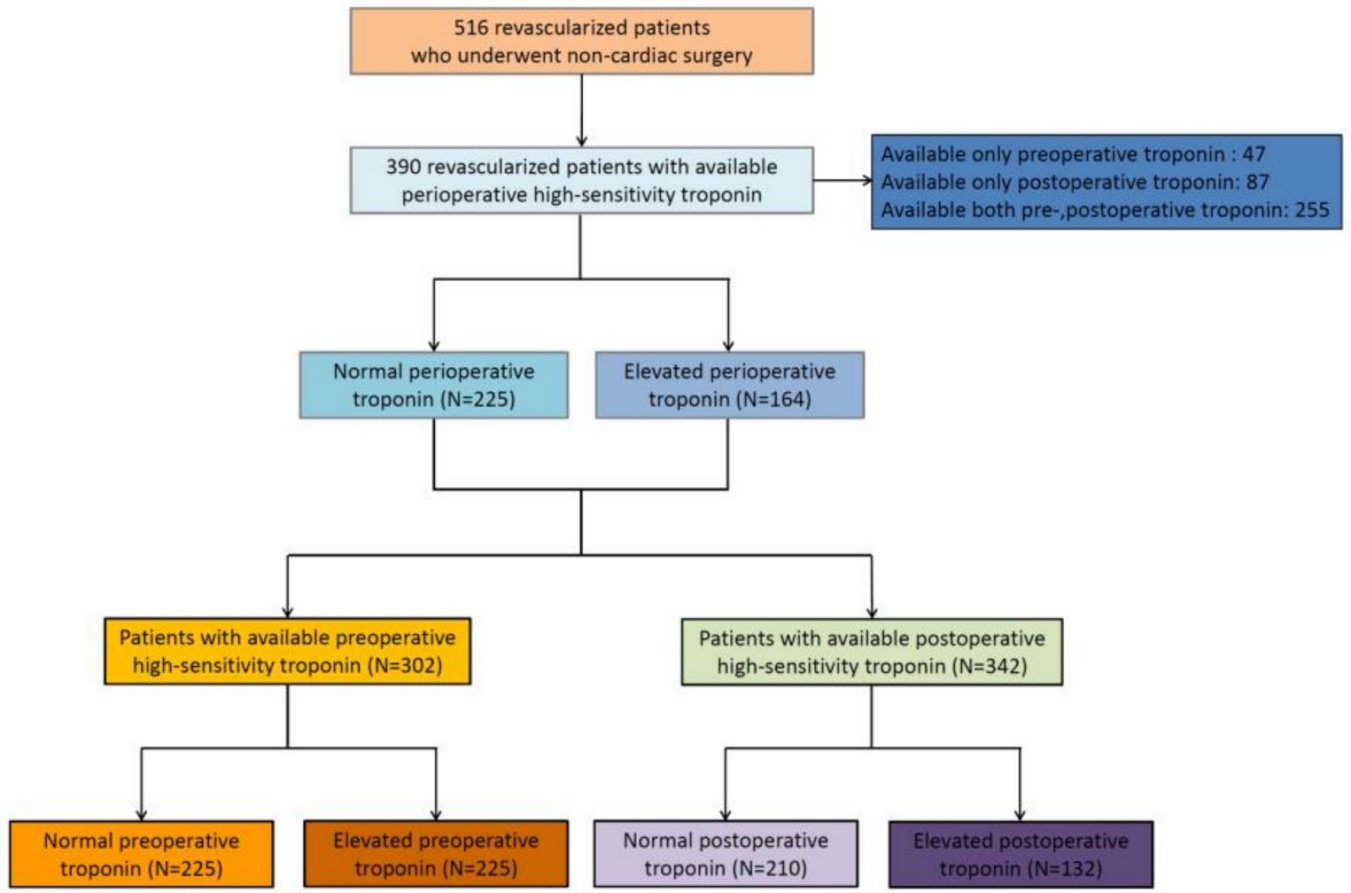
Flow chart of this study.

Hs-cTnI analysis during the perioperative period was done in a total of 389 patients. Among them, 164 patients (42.2%) showed elevated hs-cTnI during the perioperative period. We divided the patients into 2 groups according to whether the hs-cTnI assay measurements were preoperative or postoperative. Preoperative hs-cTnI was measured in 302 patients, of whom 77 showed elevated hs-cTnI (25.5%) and 225 showed normal hs-cTnI (74.5%). Postoperative hs-cTnI was measured in 342 patients, of whom 132 showed elevated hs-cTnI (38.6%) and 210 showed normal hs-cTnI (61.4%). The flow chart of this study is shown in Fig 1.

### Analysis of troponin elevation during the perioperative period

Table 1 shows the baseline and surgical characteristics of all patients. The median level of hs-cTnI was 0.135 μg/L (0.061–0.692) in the elevated group. Compared with patients in the normal hs-cTnI group, those in the elevated group had a higher rate of diabetes, chronic kidney disease, multivessel disease, ejection fraction <50%, and emergency operations and a lower use of statins. By performing propensity score matching for the entire population, we generated 142 pairs of matched datasets by 1:1 individual matching without replacement. We found no significant imbalances between the elevated and normal hs-cTnI groups in the baseline variables of the matched population (Table 1).

**Table 1:** Baseline characteristics.

	Entire population				Propensity-matched population		
	Normal troponin (N = 225)	Elevated troponin (N = 164)	*p*-value	Standardized mean difference	Normal troponin (N = 142)	Elevated troponin (N = 142)	Standardized mean difference
Male sex	169 (75.1)	120 (73.2)	0.67	-4.4	102 (71.8)	101 (71.1)	-1.6
Age (years)	69.3 ± 9.06	70.8 ± 9.35	0.13	15.2	70.5 ± 9.13	70.7 ± 9.41	1.8
Diabetes	117 (52.0)	103 (62.8)	0.03	22.3	88 (62.0)	85 (59.9)	-4.4
Hypertension	182 (80.9)	139 (84.8)	0.32	10.7	116 (81.7)	118 (83.1)	3.9
Chronic kidney disease	24 (10.7)	44 (26.8)	<0.001	36.4	22 (15.5)	27 (19.0)	7.9
History of MI	55 (24.4)	40 (24.4)	0.99	-0.01	30 (21.1)	35 (24.6)	8.2
History of stroke	32 (14.2)	30 (18.3)	0.28	10.5	25 (17.6)	23 (16.2)	-3.6
History of PAD	34 (15.1)	28 (17.1)	0.60	5.2	25 (17.6)	21 (14.8)	-7.5
CABG	105 (46.7)	82 (50.0)	0.52	6.6	67 (47.2)	65 (45.8)	-2.8
Multivessel disease	175 (77.8)	143 (87.2)	0.02	28.1	121 (85.2)	122 (85.9)	2.1
Ejection fraction <50	35 (15.6)	46 (28.0)	0.003	27.7	29 (20.4)	33 (23.2)	-6.3
Medication							
Statins	187 (83.1)	115 (70.1)	0.002	-28.3	108 (76.1)	106 (74.6)	-3.1
β-blocker	127 (56.4)	90 (54.9)	0.76	-3.1	74 (52.1)	79 (55.6)	7.1
Antiplatelet agent	191 (84.9)	141 (86.0)	0.77	3.1	124 (87.3)	121 (85.2)	-6.1
ESC/ESA risk score			0.08	14.8			0
Mild	36 (16.0)	26 (15.9)			19 (13.4)	22 (15.5)	
Moderate	141 (62.7)	87 (53.0)			84 (59.2)	78 (54.9)	
High	48 (21.3)	51 (31.1)			39 (27.5)	42 (29.6)	
Surgery type			0.23				
Vascular	67 (29.8)	47 (28.7)			47 (33.1)	40 (28.2)	
Abdominal	65 (28.9)	56 (34.1)			38 (26.8)	49 (34.5)	
Orthopedic	32 (14.2)	32 (19.5)			21 (14.8)	25 (17.6)	
Thoracic	25 (11.1)	14 (8.5)			17 (12.0)	13 (9.2)	
Neurosurgery	16 (7.1)	5 (3.0)			10 (7.0)	5 (3.5)	
ENT, ophthalmology	9 (4.0)	7 (4.3)			3 (2.1)	7 (4.9)	
Genital	11 (4.9)	3 (1.8)			6 (4.2)	3 (2.1)	
Emergency	29 (12.9)	35 (21.3)	.03	20.6	20 (14.1)	24 (16.9)	6.9
General anesthesia	207 (92.0)	155 (94.5)	.34	11.0	137 (94.4)	133 (93.7)	-3.1

Data are presented as mean ± standard deviation or n (%).

MI, myocardial infarction; PAD, peripheral artery disease; CABG, coronary artery bypass graft surgery; ESC, European Society of Cardiology; ESA, European Society of Anesthesiology; ENT, ear nose throat

**Fig 2:**
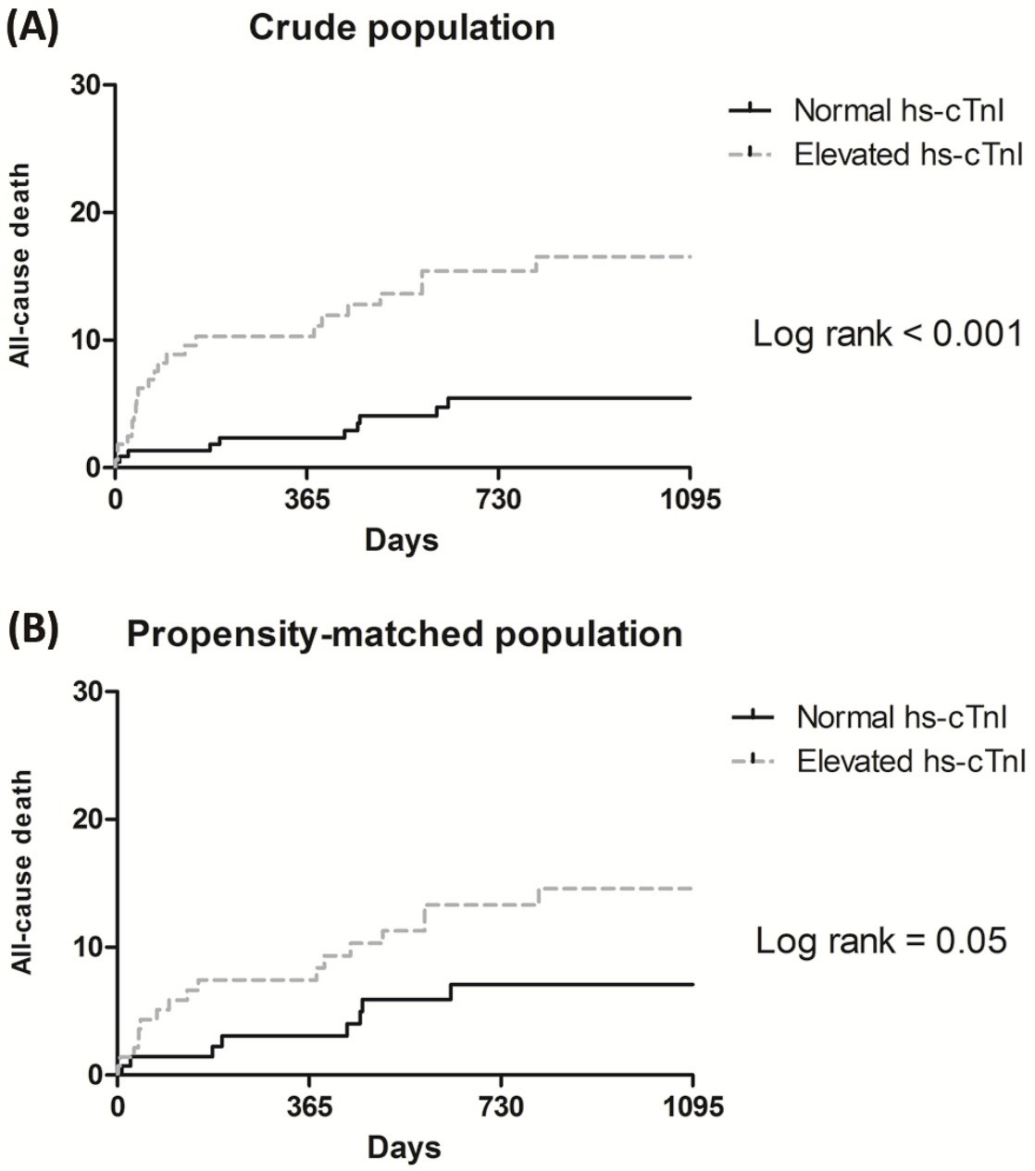
Kaplan-Meier curves for the groups in the overall population with elevated hs-cTnI and normal hs-cTnI levels. Curves for (A) the entire population and (B) the propensity-matched population.

The median follow-up duration was 25 months (IQR 15–47) in the normal hs-cTnI group and 26 months (IQR 7–48) in the elevated hs-cTnI group (*p* = 0.67). The elevated hs-cTnI group showed a higher incidence of all-cause death during follow-up in the multivariate analysis (HR, 2.97; 95% confidential interval [CI], 1.40–6.29; *p* = 0.004) (Table 2, Fig 2A). In the propensity-matched analysis, the risk of all-cause death during follow-up was similar (HR, 2.67; 95% CI, 1.04–6.82; *p* = 0.04) (Table 2, Fig 2B).

**Table 2:** All-cause death in the entire and propensity-matched populations.

					Multivariate analysis		Propensity score-matched analysis			
	Normal troponin (N = 225)	Elevated troponin (N = 164)	Unadjusted HR (95% CI)	*p*-value	Adjusted HR (95% CI)*	*p*-value	Normal troponin (N = 142)	Elevated troponin (N = 142)	Adjusted HR (95% CI)	*p*-value
All-cause death in a year	5 (2.2)	15 (9.1)	4.31 (1.57–11.85)	0.005	3.57 (1.24–10.27)	0.02	4 (2.8)	10 (7.0)	2.50 (0.78–7.97)	0.12
All-cause death	11 (4.9)	24 (14.6)	3.22 (1.58–6.58)	0.001	2.97 (1.40–6.29)	0.004	9 (6.3)	18 (12.7)	2.67 (1.04–6.82)	0.04

Data are presented as mean ± standard deviation or n (%).

HR, hazard ratio; CI, confidence interval

*Covariates were diabetes, chronic kidney disease, multivessel disease, ejection fraction <50, use of statins, and emergency surgery.

### Analysis of preoperative troponin elevation

Table 3 shows the baseline and surgical characteristics of the preoperative group. The median level of hs-cTnI was 0.063 μg/L (0.048–0.145) in the elevated hs-cTnI group. Compared with patients in the normal hs-cTnI group, those in the elevated hs-cTnI group were older and showed a higher rate of diabetes, chronic kidney disease, and ejection fraction <50% and a lower use of statins. By performing propensity score matching for the entire population, we generated 70 pairs of matched datasets by 1:N individual matching without replacement. We found no significant imbalances between the elevated and normal hs-cTnI groups in the baseline variables of the matched population (Table 3).

**Table 3:** Baseline characteristics of the preoperative groups.

	Entire population				Propensity-matched population		
	Normal troponin (N = 225)	Elevated troponin (N = 77)	*p*-value	Standardized mean difference	Normal troponin (N = 194)	Elevated troponin (N = 70)	Standardized mean difference
Male sex	172 (76.4)	51 (66.2)	0.08	-21.5	131.9 (68.0)	46 (65.7)	4.8
Age (years)	69.4 ± 9.09	70.4 ± 9.32	0.41	10.7	70.53 ± 8.64	70.53 ± 9.44	-0.05
Diabetes	121 (53.8)	55 (71.4)	0.007	50	138.2 (71.2)	48 (68.6)	-5.81
Hypertension	184 (81.8)	67 (87.0)	0.29	15.5	168.1 (86.7)	61 (87.1)	1.41
Chronic kidney disease	49 (21.8)	29 (37.7)	0.006	32.6	61.6 (31.8)	24 (34.3)	5.17
History of MI	54 (24.0)	21 (27.3)	0.57	7.3	48.6 (25.1)	19 (27.1)	4.62
History of stroke	39 (17.3)	13 (16.9)	0.93	-1.19	34.9 (18.0)	12 (17.1)	-2.21
History of PAD	36 (16.0)	18 (23.4)	0.15	17.3	42.7 (22.0)	17 (24.3)	5.31
CABG	109 (48.4)	41 (53.2)	0.47	9.56	92.8 (47.8)	35 (50.0)	4.31
Multivessel disease							
Ejection fraction <50	40 (17.8)	24 (31.2)	0.01	28.7	50.1 (25.8)	19 (27.1)	2.86
Medication							
Statins	180 (80.0)	52 (67.5)	0.03	-26.5	145.6 (75.0)	52 (74.3)	-1.62
β-blocker	132 (58.7)	43 (55.8)	0.67	-5.65	108.6 (56.0)	39 (55.7)	-0.52
Antiplatelet agent	177 (78.7)	54 (70.1)	0.13	-18.5	156.7 (80.8)	58 (82.9)	5.34
ESC/ESA risk score			0.15				
Mild	35 (15.6)	18 (23.4)		18.4	38.2 (19.7)	14 (20.0)	0.56
Moderate	135 (60.0)	37 (48.1)		-23.7	103.0 (53.1)	22 (31.4)	-8.95
High	55 (24.4)	22 (28.6)		9.08	52.8 (27.2)	22 (31.4)	9.27
Surgery type			0.23				
Vascular	82 (36.4)	21 (27.3)			69 (35.6)	20 (28.6)	
Abdominal	64 (28.4)	24 (31.2)			53 (27.3)	21 (30.0)	
Orthopedic	33 (14.7)	15 (19.5)			29 (14.9)	13 (18.6)	
Thoracic	18 (8.0)	6 (7.8)			15 (7.7)	6 (8.6)	
Neurosurgery	13 (5.8)	3 (3.9)			13 (6.7)	3 (4.3)	
ENT, ophthalmology	5 (2.2)	6 (7.8)			5 (2.6)	6 (8.6)	
Genital	10 (4.4)	2 (2.6)			10 (5.2)	1 (1.4)	
Emergency	37 (16.4)	18 (23.4)	0.17	16.3	34.0 (17.5)	14 (20.0)	0.98
General anesthesia	212 (94.2)	72 (93.5)	0.82	-2.89	183.4 (94.5)	66 (94.3)	0.54

Data are presented as mean ± standard deviation or n (%).

MI, myocardial infarction; PAD, peripheral artery disease; CABG, coronary artery bypass graft surgery; ESC, European Society of Cardiology; ESA, European Society of Anesthesiology; ENT, ear nose throat

**Fig 3:**
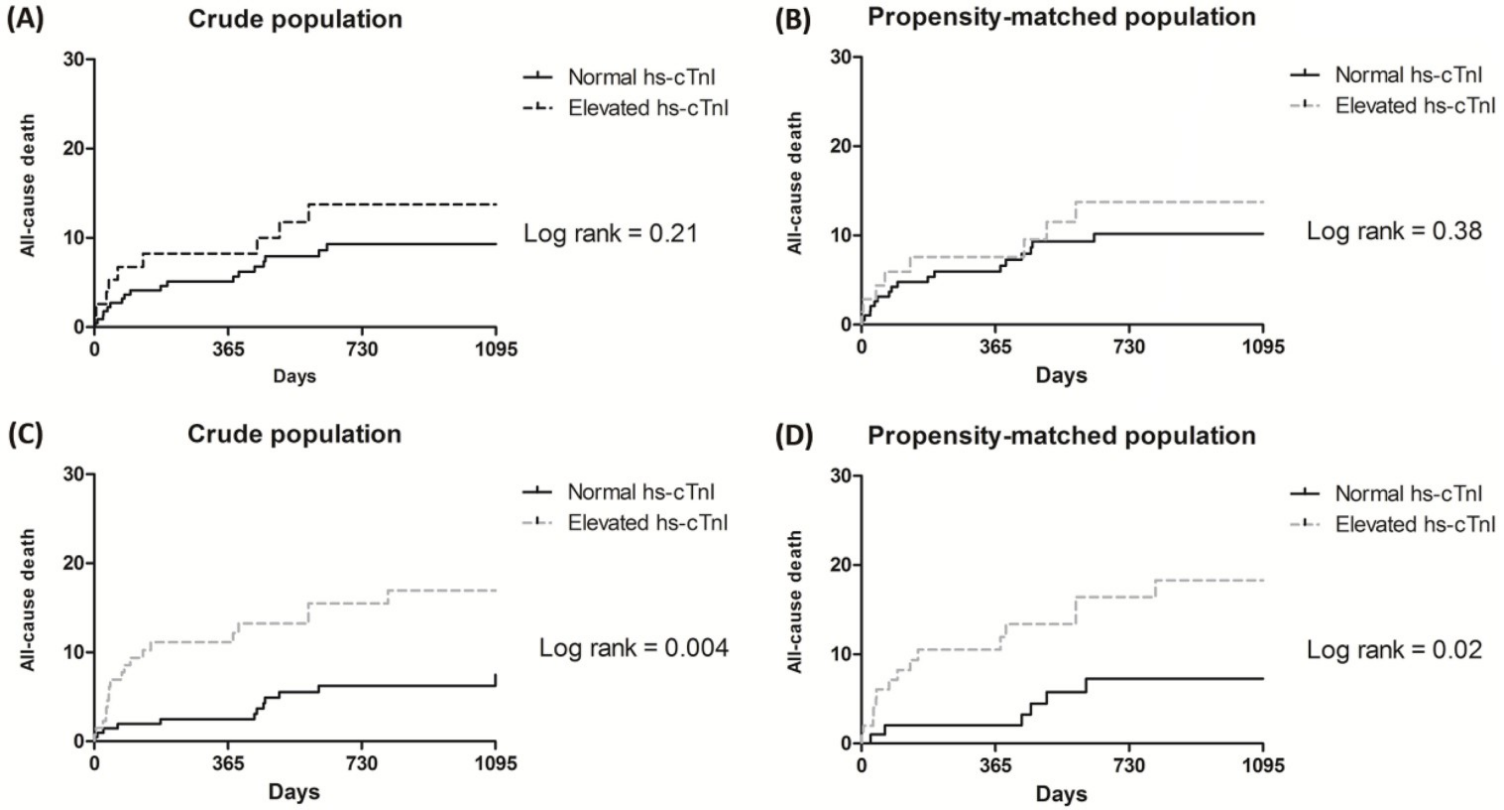
Kaplan-Meier curves for the groups with elevated hs-cTnI and normal hs-cTnI in pre- and postoperative analyses. Curves for (A) the entire preoperative population, (B) the propensity-matched preoperative population, (C) the entire postoperative population, and (D) the propensity-matched postoperative population.

The median follow-up duration was 24 months (IQR 13–45) in the group with normal hs-cTnI and 25 months (IQR 8–49) in the group with elevated hs-cTnI (*p* = 0.92). The incidence of all-cause death during follow-up did not differ between patients in the normal and elevated hs-cTnI groups in the multivariate analysis (HR, 0.79; 95% CI, 0.25–2.48; *p* = 0.68) (Table 4, Fig 3A). In the propensity-matched analysis, the risk of all-cause death during follow-up was similar (HR, 1.06; 95%CI, 0.45–2.50; *p* = 0.89) (Table 4, Fig 3B).

**Table 4:** All-cause death in the preoperative group (Entire and propensity-matched populations).

					Multivariate analysis		Propensity score-matched analysis			
	Normal troponin (N = 225)	Elevated troponin (N = 77)	Unadjusted HR (95% CI)	*p*-value	Adjusted HR (95% CI)*	*p*-value	Normal troponin (N = 194)	Elevated troponin (N = 70)	Adjusted HR (95% CI)	*p*-value
All-cause death in a year	10 (8.9)	6 (11.7)	1.37 (0.63–3.02)	0.41	1.03 (0.44–2.38)	0.95	14.6 (7.7)	5 (7.1)	0.96 (0.32–2.82)	0.94
All-cause death	19 (5.3)	10 (6.5)	1.26 (0.44–3.56)	0.67	0.79 (0.25–2.48)	0.68	23.2 (11.9)	9 (12.9)	1.06 (0.45–2.50)	0.89

Data are presented as mean ± standard deviation or n (%).

HR, hazard ratio; CI, confidence interval

*Covariates were being male; having diabetes, chronic kidney disease, or an ejection fraction <50; and using statins.

### Analysis of postoperative troponin elevation

Table 5 shows the baseline and surgical characteristics of the postoperative group. The median level of hs-cTnI was 0.153 μg/L (0.063–0.709) in the group with elevated hs-cTnI. Compared with patients in the normal hs-cTnI group, those in the elevated hs-cTnI group showed a higher rate of diabetes, chronic kidney disease, multivessel disease, and ejection fraction <50% and a lower use of statins. By performing propensity score matching for the entire population, we generated 101 pairs of matched datasets by 1:1 individual matching without replacement. We found no significant imbalances in the baseline variables of the matched population between patients in the elevated and normal hs-cTnI groups (Table 5).

**Table 5:** Baseline characteristics of the postoperative group.

	Entire population				Propensity-matched population		
	Normal troponin (N = 210)	Elevated troponin (N = 132)	*p*-value	Standardized mean difference	Normal troponin (N = 101)	Elevated troponin (N = 101)	Standardized mean difference
Male sex	160 (76.2)	97 (73.5)	0.57	-6.1	76 (75.2)	77 (76.2)	-2.2
Age (years)	69.8 ± 9.05	70.7 ± 9.28	0.37	9.8	70.06 ± 8.58	69.91 ± 9.06	-1.6
Diabetes	106 (50.5)	82 (62.1)	0.04	23.9	59 (58.4)	56 (55.4)	6.1
Hypertension	169 (80.5)	111 (84.1)	0.40	9.8	84 (83.2)	83 (82.2)	2.7
Chronic kidney disease	14 (6.7)	30 (22.7)	<0.001	38.2	12 (11.9)	11 (10.9)	2.4
History of MI	50 (23.8)	33 (25.0)	0.80	2.7	24 (23.8)	21 (20.8)	6.8
History of stroke	30 (14.3)	25 (18.9)	0.25	11.8	18 (17.8)	18 (17.8)	0
History of PAD	34 (16.2)	19 (14.4)	0.66	-5.1	15 (14.9)	14 (13.9)	2.8
CABG	105 (50.0)	65 (49.2)	0.89	-1.5	49 (48.5)	50 (49.5)	-2.0
Multivessel disease	161 (76.7)	117 (88.6)	0.006	37.6	89 (88.1)	91 (90.1)	-6.2
Ejection fraction <50	33 (15.7)	40 (30.3)	0.001	31.6	23 (22.8)	21 (20.8)	4.3
Medication							
Statins	173 (82.4)	94 (71.2)	0.02	-24.6	78 (77.2)	78 (77.2)	0
β-blocker	118 (56.2)	74 (56.1)	0.98	-0.3	57 (56.4)	55 (54.5)	4.0
Antiplatelet agent	179 (85.2)	116 (87.9)	0.49	8.1	90 (89.1)	89 (88.1)	3.0
ESC/ESA risk score			0.14	19.2			-9.9
Mild	29 (13.8)	15 (11.4)			8 (7.9)	12 (11.9)	
Moderate	130 (61.9)	72 (54.5)			59 (58.4)	58 (57.4)	
High	51 (24.3)	45 (34.1)			34 (33.7)	31 (30.7)	
Surgery type			0.23				
Vascular	67 (31.9)	39 (29.5)			35 (34.7)	28 (27.7)	
Abdominal	69 (32.9)	44 (33.3)			34 (33.7)	37 (36.6)	
Orthopedic	27 (12.9)	28 (21.2)			10 (9.9)	18 (17.8)	
Thoracic	18 (8.6)	12 (9.1)			10 (9.9)	12 (11.9)	
Neurosurgery	15 (7.1)	3 (2.3)			8 (7.9)	2 (2.0)	
ENT, ophthalmology	8 (3.8)	4 (3.0)			1 (1.0)	3 (3.0)	
Genital	6 (2.9)	2 (1.5)			3 (3.0)	1 (1.0)	
Emergency	30 (14.3)	27 (20.5)	0.14	15.2	15 (14.9)	19 (18.8)	9.8
General anesthesia	197 (93.8)	125 (94.7)	0.73	3.9	95 (94.1)	96 (95.0)	4.4

Data are presented as mean ± standard deviation or n (%).

MI, myocardial infarction; PAD, peripheral artery disease; CABG, coronary artery bypass graft surgery; ESC, European Society of Cardiology; ESA, European Society of Anesthesiology

The median follow-up duration was 27 months (IQR 15–50) in the normal hs-cTnI group and 25 months (IQR 6–45) in the elevated hs-cTnI group. Patients in the elevated hs-cTnI group showed a higher rate of all-cause death in the multivariate analysis (HR, 2.36; 95% CI, 1.10–5.05; *p* = 0.006) (Table 6, Fig 3C). In the multivariate analysis, the risk of all-cause death during 1 year was also higher in those patients with elevated hs-cTnI (HR, 3.61; 95% CI, 1.25–10.43; *p* = 0.003). In the propensity-matched analysis, the rate of all-cause death was higher in the group with elevated hs-cTnI (HR, 2.80; 95%CI 1.01–7.77; *p* = 0.048) (Table 6, Fig 3D). In addition, all-cause death in a year was higher in the group with elevated hs-cTnI (HR, 5.00; 95%CI 1.10–22.83; *p* = 0.038) (Table 6). Among the patients with PMI, 5 patients underwent coronary angiograms, 4 patients were treated with PCI (two by stent thrombosis and two by non-target vessel revascularization), and 1 patient was diagnosed with stress-induced cardiomyopathy.

**Table 6:** All-cause death in the postoperative group (entire and propensity-matched populations).

					Multivariate analysis		Propensity score-matched analysis			
	Normal troponin (N = 210)	Elevated troponin (N = 132)	Unadjusted HR (95% CI)	*p*-value	Adjusted HR (95% CI)*	*p*-value	Normal troponin (N = 101)	Elevated troponin (N = 101)	Adjusted HR (95% CI)	*p*-value
All-cause death in a year	5 (2.4)	14 (10.6)	4.62 (1.66–12.83)	0.003	3.61 (1.25–10.43)	0.018	2 (2.0)	10 (9.9)	5.00 (1.10–22.83)	0.038
All-cause death	12 (5.7)	19 (14.4)	2.76 (1.34–5.69)	0.006	2.36 (1.10–5.05)	0.027	6 (5.9)	15 (14.9)	2.80 (1.01–7.77)	0.048

Data are presented as mean ± standard deviation or n (%).

HR, hazard ratio; CI, confidence interval

*Covariates were diabetes, chronic kidney disease, multivessel disease, ejection fraction <50, and use of statins.

## Discussion

The main findings of this study are as follows: 1) hs-cTnI elevation during the perioperative period is associated with all-cause mortality in revascularized patients, and 2) postoperative elevation of hs-cTnI (but not preoperative) showed an association with all-cause death in revascularized coronary patients who underwent noncardiac surgery. This finding indicates that postoperative troponin analysis is important for evaluating the prognosis of revascularized coronary patients undergoing noncardiac surgery. Contrarily, preoperative hs-cTnI levels do not appear to be a helpful predictor of prognosis in those patients.

### Perioperative troponin level and clinical outcomes

In the general population, elevated basal troponin shows robust associations with an increased risk of cardiovascular and all-cause mortality [17,18]. Even in patients with stable coronary artery disease, basal cardiac troponin elevation is associated with the incidence of cardiovascular disease and heart failure [14]. Several trials have shown an association between preoperative troponin elevation and short- or long-term mortality in patients undergoing noncardiac surgery. Preoperative troponin was associated with a higher than usual rate of major adverse cardiac events in vascular surgery patients [11]. Emergency surgery under general anesthesia with preoperative troponin elevation carries an increased risk of postoperative adverse events, including death [12]. Moreover, a previous study suggested that delaying surgery appeared to reduce the risk for patients with preoperative troponin elevation [6]. In addition, postoperative troponin elevation has a strong association with all-cause mortality [9,10]. Driven by these results, troponin screening is recommended as a routine measure for surgical inpatients [19]. However, this hypothesis had not been fully evaluated in patients with a history of coronary revascularization. In the present study, we found that troponin elevation during the perioperative period (pre- and postoperative) was associated with all-cause mortality in revascularized coronary patients, which correlates well with prior studies. Moreover, we found that the analysis produced more subtle findings when patients were divided according to the time when troponin was measured.

### Preoperative troponin elevation in revascularized patients

We found that preoperative hs-cTnI elevation was not associated with all-cause death during follow-up in revascularized patients who underwent noncardiac surgery. We offer several explanations for this result. First, the 99^th^ percentile value of troponin in revascularized patients might differ from that in healthy subjects. A prior study that targeted patients with stable coronary artery disease showed that the incidence of subjects above the 99^th^ percentile value of cardiac troponin T was 11.1% [14]. We, on the other hand, found an incidence above 25%. The current evidence does not support the use of a universal definition in optimal criterion to identify clinically relevant post-PCI myocardial infarction (MI) events in patients undergoing PCI [20]. Therefore, it might not be appropriate to apply the 99^th^ percentile value of troponin to patients who have already received revascularization via either PCI or CABG. Second, the increased sensitivity of the troponin assay could affect the result. Advanced assay techniques have lowered the 99^th^ percentile value, which has adversely affected clinical specificity; the currently available high-sensitivity troponin assay has a low specificity for MI [21,22]. The difficulty that such low specificity causes in determining when troponin elevation is accompanied by myocardial ischemia could be exaggerated in revascularized coronary patients. Finally, stressful conditions associated with noncardiac surgery might also have affected our results. Myocardial ischemia during stress is associated with higher resting levels of hs-cTnI in patients with coronary artery disease [23]. However, it is unclear whether the association between hs-cTnI and adverse outcomes is caused by the presence of myocardial ischemia in these patients. The association between an elevated resting hs-cTnI level and adverse outcomes in patients treated with coronary revascularization needs more investigation. Consequently, it is reasonable for clinicians to be cautious when using the troponin assay as a screening test for risk stratification before noncardiac surgery, and an alternative upper limit to the normal value is needed for specific patients, such as those with a history of coronary revascularization, who might have a higher basal troponin level than the normal population.

### PMI after noncardiac surgery in revascularized coronary patients

PMI after noncardiac surgery, also known as myocardial injury after noncardiac surgery (MINS), is an important prognostic factor in patients who undergo noncardiac surgery [7–9,11]. However, that hypothesis has not been fully evaluated in patients with a history of coronary revascularization. In the present study, the prevalence of patients with PMI was higher (38.6%) than in previous studies, with only 4 patients diagnosed with coronary occlusion. The VISION study (Vascular Events in Noncardiac Surgery Patients Cohort Evaluation) showed that the rate of PMI after noncardiac surgery was 17.9%, and another study showed it to be 16% [9,10]. The high rate of PMI after noncardiac surgery in the present study could be explained as follows. First, coronary patients revascularized by either PCI or CABG are more vulnerable to secondary ischemia than the normal population. The dominant mechanism of PMI is actually a supply–demand mismatch attributable to hypotension, anemia, and tachycardia rather than plaque rupture [24]. Therefore, the rate of type II MI occurrence is higher in those patients, which might have affected our results [25,26]. Second, the definition of PMI in our study might have caused a higher prevalence. We defined PMI as above the 99^th^ percentile of hs-cTnI irrespective of the preoperative hs-cTnI value, so patients with preoperative troponin elevation might have been included.

The results in the present study indicate that PMI after noncardiac surgery is associated with all-cause mortality in revascularized coronary patients, whereas patients with preoperative troponin elevation do not show that association. We explain this distinction in several ways. First, the increment level of hs-cTnI was different between the pre- and postoperative test. The median level of postoperative troponin in patients with PMI was almost 2.5-fold higher than in patients with elevated preoperative troponin levels. Such an increase in the maximum concentrations of postoperative troponin measured using the high-sensitivity assay was previously associated with an increasing mortality rate [9]. In addition, the cause of troponin elevation differs between the pre- and postoperative periods. Most preoperative myocardial injury is caused by chronic diseases, although the mechanism of myocardial injury varies [27]. In the case of PMI after noncardiac surgery, we assume that the main cause of PMI is an acute decompensated condition following noncardiac surgery. In most patients with PMI, the myocardial injury seems to be caused by a supply–demand mismatch attributable to hypotension, anemia, and tachycardia rather than plaque rupture [9,24]. Changes caused by these conditions could lead to higher mortality. Another possible explanation is that patients with PMI might be particularly vulnerable to ischemic injury, which could lead to increased mortality; however, that possibility requires further investigation. According to our results, a postoperative check for high-sensitivity troponin might be important for predicting the prognosis of patients with a history of coronary revascularization.

### Statin and troponin level

One intriguing finding of this study is that patients with PMI used less statin medication than the other patients in our population. One randomized study showed that statin therapy in moderate hypercholesterolemia patients reduced troponin concentration by 13% compared to the placebo group, and the group taking statins also had decreased MI and cardiac death compared with the placebo group [28]. Our data are similar, indicating that a clinical investigation or randomized study of the association between preoperative statin therapy and the prevention of PMI might be needed in patients with a history of coronary revascularization undergoing noncardiac surgery.

### Current status of the high-sensitivity cardiac troponin assay

Rapid advances in immunoassay technologies and the international adoption of traceable troponin calibration standards have allowed manufacturers to develop and calibrate troponin assays with unprecedented analytic sensitivity and accuracy [15]. Therefore, troponin testing is now used as a screening test in a broad spectrum of non-acute coronary syndrome patients, including patients with end-stage renal disease, sepsis, and congestive heart failure, which are conditions that are all associated with elevated troponin levels [21]. However, there are some obstacles to the broad use of high-sensitivity troponin assays. First, using a test in a broad unselected group of patients can result in a phenomenon known as *spectrum bias* [21]. In other words, use of the troponin assay in a normal population can decrease the specificity of the test. Second, limited data are available about the cost-effectiveness of such screening [29]. Third, it remains unclear whether the implementation of high-sensitivity assays will cause an increase in testing and inappropriate therapies, which could increase the resources used for cardiology consultations and admissions for acute myocardial injury without an acute coronary syndrome [29]. According to the results of this study, routine checks of preoperative troponin levels might not be helpful, though a postoperative troponin analysis could help to predict postoperative mortality in revascularized coronary patients. Therefore, clinicians should understand the operating characteristics and basic analytical concepts of the high-sensitivity troponin assay to ensure that they use and interpret its results appropriately.

### Limitations

Our results should be considered in light of the following limitations. This study was not randomized, and therefore potential confounding factors or selection bias might have significantly affected the results. There is no universally accepted definition of PMI. We did not pre-specify the treatment strategy, stenting, or surgical technique. Another limitation is an insufficient amount of detailed medical information about patients whose coronary revascularizations were performed outside of our center. Although their records were transferred on their former medical chart, detailed information such as the vessel number, stent type, and complete revascularization was not available for every patient. Advances in devices, techniques, and operator experience during the study period are not reflected in our results. The misclassification of chronic hs-cTnI elevations as PMI might have occurred because not all patients received both pre-and postoperative troponin analyses. Despite the limitations, this study provides rigorous propensity-matched data on the clinical usefulness of preoperative troponin levels, so it can inform current practices of risk evaluation in patients undergoing noncardiac surgery.

## Conclusion

In coronary patients revascularized by CABG or PCI, a postoperative hs-cTnI evaluation might be helpful in evaluating prognosis after noncardiac surgery. Analyses of larger registry datasets are needed to support this finding.

## Supporting information

S1 File Dataset for PO. (CSV)
